# Supplementary material for: Numerical simulations of a kilometre-thick Arctic ice shelf consistent with ice grounding observations
Source: Nat Commun. 2018 Apr 17;9:1510. doi: 10.1038/s41467-018-03707-w (PMC5904099; doi:10.1038/s41467-018-03707-w)
Supplement: Supplementary file 1 — Supplementary Information(PDF 8436 kb) [file 41467_2018_3707_MOESM1_ESM.pdf]

Supplementary Information

Numerical simulations of a kilometre-thick Arctic ice shelf consistent with ice grounding observations

Gasson et al., 2018

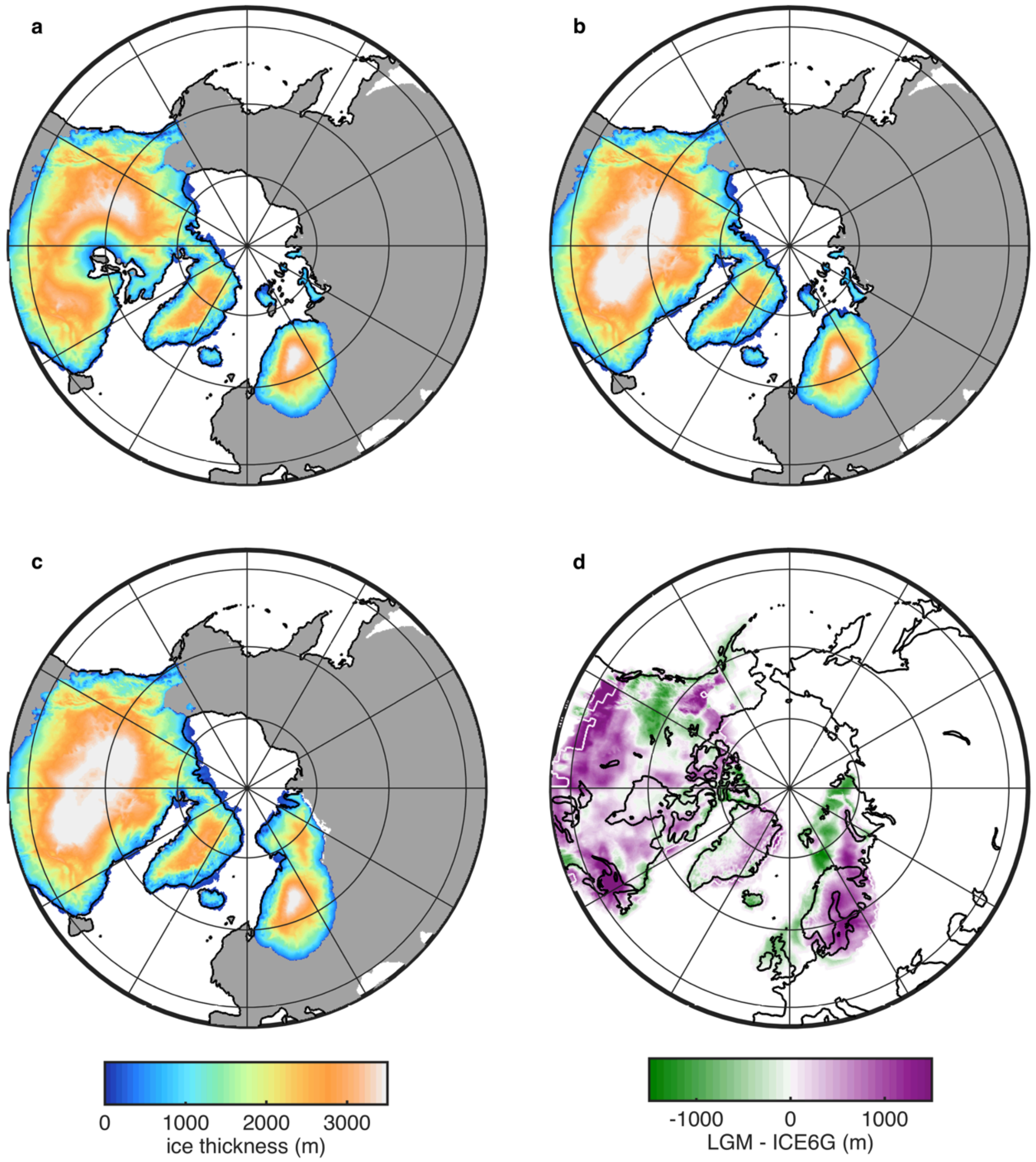

Supplementary Figure 1 – LGM control simulations with different calving thresholds. Minimum ice shelf thickness calving threshold, see equation B.5 in ref [1], equal to (**a**) 150 m (default), (**b**) 125 m, or (**c**) 100 m. Note that calving is too aggressive and prevents the formation of the Barents-Kara ice sheet with a calving thickness threshold of 150-125 m, the ice sheet also cannot expand across Hudson Bay with a calving threshold of 150 m. Simulations use an LGM climate forcing[2]. **d**, ice thickness anomaly for simulation (**c**), relative to ICE-6G for the LGM. Ice shelves are prevented from forming in the Arctic in these LGM simulations. Gray shading is land above sea level, expanded from modern largely due to lowering of sea level.

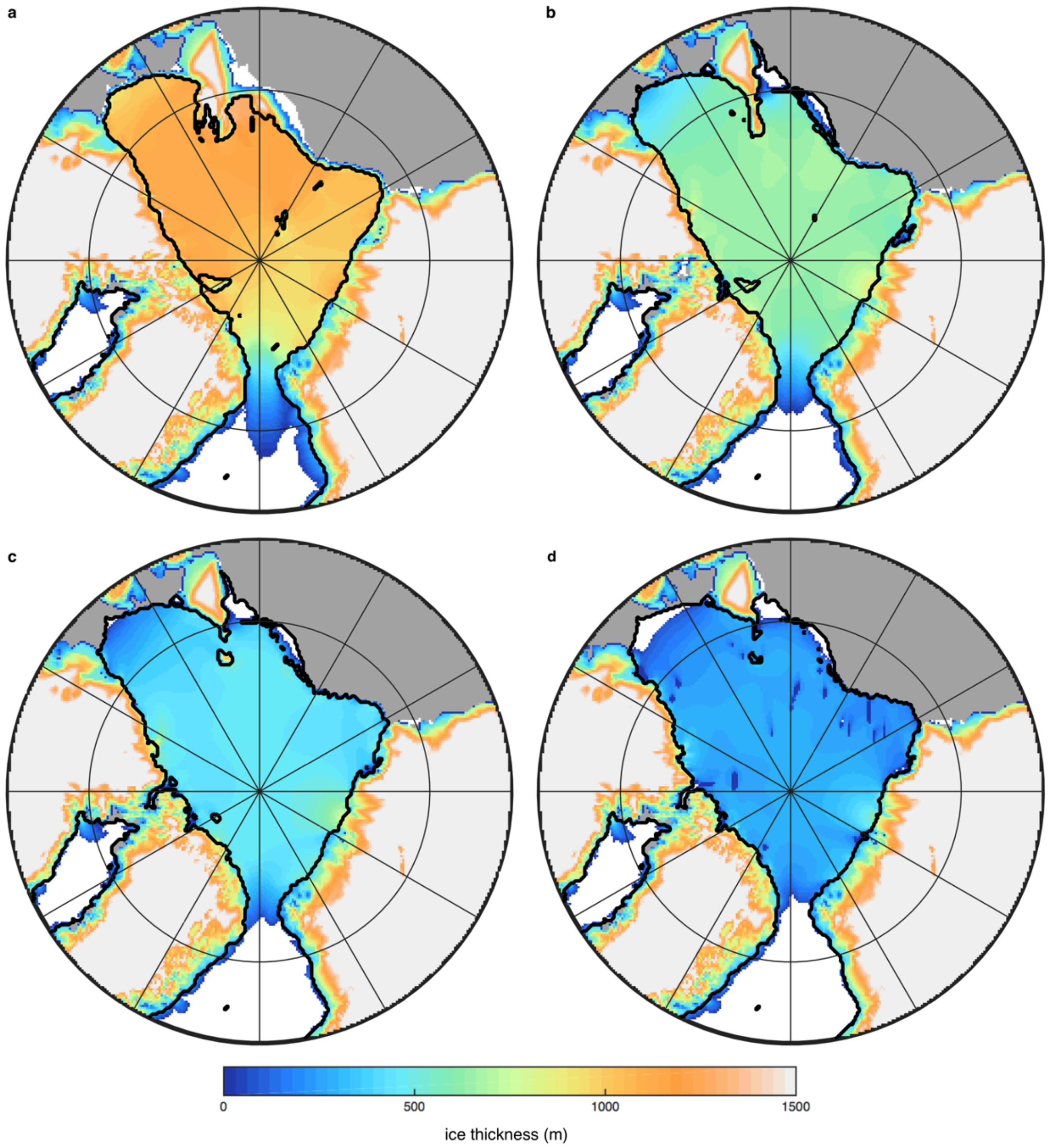

Supplementary Figure 2 – Ocean melt sensitivity test. Tests performed for simulation equivalent to Fig 2a in the main text, for ocean melt enhancement factors of 0.5, 2, 4 and 8 for **a-d**. The equivalent ocean melt rate at 1000 m depth in the central Arctic Ocean is 0.08, 0.32, 0.64 and 1.27 m yr^{-1}, for **a-d**. Ocean melt rates are calculated from modeled ocean temperatures[3].

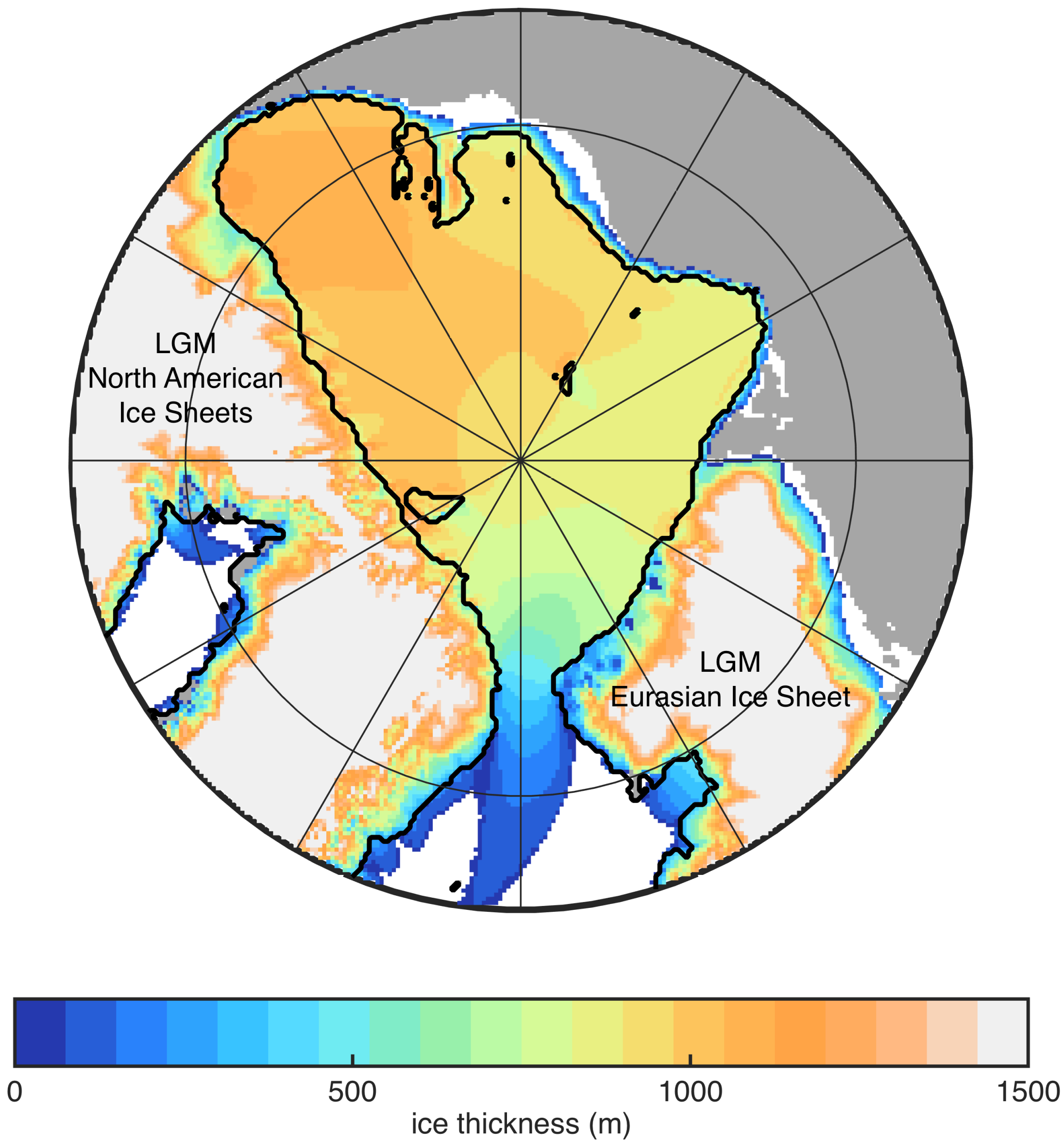

Supplementary Figure 3 – Simulation with LGM North American and Eurasian ice sheets – Climate forcing from Ref [2], with exception of ice sheet extent and climate input, simulations are identical to MIS6 simulations. Mean ice shelf thickness 940 m.

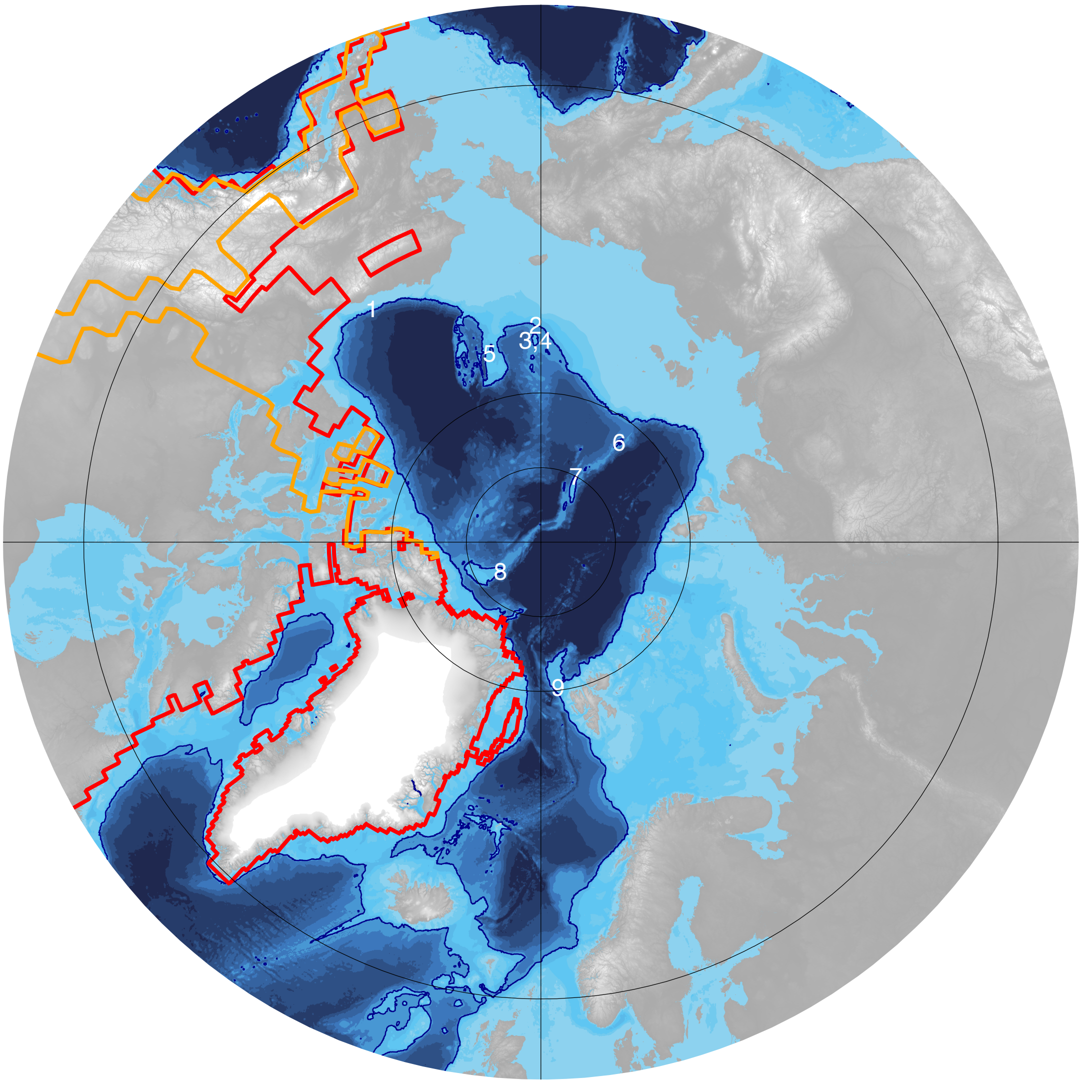

Supplementary Figure 4 – Location of ice grounding evidence and North American Ice Sheet outlines. Numbers show locations of ice grounding evidence shown in Table 1. North American ice sheet extent from ICE5G[4], which is used as input into the climate model simulations[2,5], for the LGM (red line) and 13 ka (orange line).

1. Pollard, D., DeConto, R. M. & Alley, R. B. Potential Antarctic Ice Sheet retreat driven by hydrofracturing and ice cliff failure. *Earth Planet. Sci. Lett.* **412,** 112–121 (2015).
2. Brady, E. C., Otto-bliesner, B. L., Kay, J. E. & Rosenbloom, N. Sensitivity to glacial forcing in the CCSM4. *J. Clim.* **26,** 1901–1925 (2013).
3. Liu, Z. *et al.* Transient simulation of last deglaciation with a new mechanism for Bolling-Allerod warming. *Science (80-.).* **325,** 310–4 (2009).
4. Peltier, W. R. Global glacial isostasy and the surface of the ice - age Earth: The ICE - 5G (VM2) model and GRACE. *Annu. Rev. Earth Planet. Sci.* **32,** 111–149 (2004).
5. Colleoni, F., Wekerle, C., Naslund, J. O., Brandefelt, J. & Masina, S. Constraint on the penultimate glacial maximum Northern Hemisphere ice topography (~140 kyrs BP). *Quat. Sci. Rev.* **137,** 97–112 (2016).
