# Supplementary material for: Numerical simulations of a kilometre-thick Arctic ice shelf consistent with ice grounding observations
Source: Nat Commun. 2018 Apr 17;9:1510. doi: 10.1038/s41467-018-03707-w (PMC5904099; doi:10.1038/s41467-018-03707-w)
Supplement: Supplementary file 3 — Description of Additional Supplementary Files(PDF 68 kb) [file 41467_2018_3707_MOESM3_ESM.pdf]

Description of Additional Supplementary Files

File Name: Supplementary Movie 1
Description: **Ice shelf inception**. For simulation shown in Fig. 3b, showing ice shelves from Laurentide and Eurasian ice sheets coalescing in the central Arctic. Model time (yr) is shown.
